# Plant-Based Synthesis of Zinc Oxide Nanoparticles (ZnO-NPs) Using Aqueous Leaf Extract of *Aquilegia pubiflora*: Their Antiproliferative Activity against HepG2 Cells Inducing Reactive Oxygen Species and Other *In Vitro* Properties

**DOI:** 10.1155/2021/4786227

**Published:** 2021-08-17

**Authors:** Hasnain Jan, Muzamil Shah, Anisa Andleeb, Shah Faisal, Aishma Khattak, Muhammad Rizwan, Samantha Drouet, Christophe Hano, Bilal Haider Abbasi

**Affiliations:** ^1^Department of Biotechnology, Quaid-i-Azam University, Islamabad 45320, Pakistan; ^2^Institute of Biotechnology and Microbiology, Bacha Khan University, KPK, Pakistan; ^3^Department of Bioinformatics, Shaheed Benazir University Peshawar, KPK, Pakistan; ^4^Centre for Biotechnology and Microbiology, University of Swat, KPK, Pakistan; ^5^Laboratoire de Biologie des Ligneux et des Grandes Cultures (LBLGC), INRA USC1328 Université ď Orléans, Cedex 2, France

## Abstract

The anti-cancer, anti-aging, anti-inflammatory, antioxidant, and anti-diabetic effects of zinc oxide nanoparticles (ZnO-NPs) produced from aqueous leaf extract of *Aquilegia pubiflora* were evaluated in this study. Several methods were used to characterize ZnO-NPs, including SEM, FTIR, XRD, DLS, PL, Raman, and HPLC. The nanoparticles that had a size of 34.23 nm as well as a strong aqueous dispersion potential were highly pure, spherical or elliptical in form, and had a mean size of 34.23 nm. According to FTIR and HPLC studies, the flavonoids and hydroxycinnamic acid derivatives were successfully capped. Synthesized ZnO-NPs in water have a zeta potential of -18.4 mV, showing that they are stable solutions. The ZnO-NPs proved to be highly toxic for the HepG2 cell line and showed a reduced cell viability of 23.68 ± 2.1% after 24 hours of ZnO-NP treatment. ZnO-NPs also showed excellent inhibitory potential against the enzymes acetylcholinesterase (IC_50_: 102 *μ*g/mL) and butyrylcholinesterase (IC_50_: 125 *μ*g/mL) which are involved in Alzheimer's disease. Overall, the enzymes involved in aging, diabetes, and inflammation showed a moderate inhibitory response to ZnO-NPs. Given these findings, these biosynthesized ZnO-NPs could be a good option for the cure of deadly diseases such as cancer, diabetes, Alzheimer's, and other inflammatory diseases due to their strong anticancer potential and efficient antioxidant properties.

## 1. Introduction

Nanotechnology is an interdisciplinary science that encompasses several disciplines, including electronics, biomaterials, and medicine. A number of techniques, including physical, chemical, and biological processes, can be used to create nanomaterials with useful characteristics as tiny as 10–100 nm in size [1, 2]. Because of their large surface area, small size, thermal conductivity, shape, surface morphology, charge, zeta potential, and crystal structure [3], nanoscale materials have piqued the interest of scientists, allowing them to be integrated into biotechnological and biomedical sectors, particularly for the cure of deadly diseases, i.e., cancer and Alzheimer's [4, 5]. Traditional methods (chemical and physical) for producing nanoparticles have numerous limitations [6], including long-term processing, high prices, tedious procedures, hazardous by-products, and in particular the usage of poisonous chemicals [7]. However, due of its cost effectiveness, environmental friendliness, biocompatibility, convenience of use, and quick synthesis procedures, green synthesis is a favored technique for nanoparticle production [8, 9]. NPs can be biosynthesized by a variety of biological entities, including cyanobacteria, fungus, actinomycetes, bacteria, algae, and plants. Green-synthesized nanoparticles offer a new perspective as a delivery vehicle, for specific and safer drug delivery, a promising alternative to cancer drugs. Several NPs have been synthesized by green synthesis, such as Ag, Cu, Au, ZnO, Se, and CuO, and many others that have unique biological activities [10–13].

Due to its multiple uses in several technical sectors, metal oxide NPs have been actively investigated during the last decade. ZnO-NPs are an interesting inorganic material with a varied series of uses in a variety of fields, including semiconductors, energy conservation, textiles, cosmetics, electronics, health care, catalysis, and chemical sensors [14–16]. ZnO-NPs are nontoxic, biocompatible, and cheap and have a wide range of biological uses, including targeted drug delivery, anti-inflammatory, wound healing, antimicrobial agents, anti-cancer, and bioimaging [17]. ZnO-NPs are also utilized in beauty care products and sunscreens due to their effective UV absorption capabilities [18]. The use of ZnO-NPs as additives in nutritional products was certified a few years ago to increase growth performance, improve antioxidant properties, and boost the quality of eggs and chickens [19].

Metal oxide (MNPs) may be produced in a variety of ways (chemical, physical, and biosynthetic) and have a diverse set of characteristics and uses. Green synthesis covers the synthesis from algae, fungus, plants, bacteria, and other microorganisms. They enable the large-scale manufacturing of ZnO-NPs devoid of contaminants [20]. Plant parts including the leaf, stem, root, fruit, and seed have been utilized to generate ZnO-NPs because of the specific phytochemicals they produce. Natural extracts of plant components offer a low-cost and environmentally beneficial alternative to using intermediary base groups [21]. Secondary plant compounds found in plant extracts function as both reducing agents and capping or stabilizing agents. Metal ions or metal oxides are reduced to zero valence metal NPs in bioreduction with the aid of plant-secreted phytochemicals such as polyphenolic compounds, alkaloids, polysaccharides, amino acids, vitamins, and terpenoids [20]. Plants of the *Lamiaceae* family such as *Vitex negundo*, *Plectranthus amboinicus*, and *Anisochilus carnosus* have been extensively researched, revealing NP production in a variety of sizes and forms, including rod-shaped, hexagonal, quasispherical, and spherical with agglomerates. The results clearly showed that the size of produced NPs reduces as the content of a plant extract increases. For the most part, the leaves of *A. indica* of the *Meliaceae* family have been utilized in the synthesis of ZnO-NPs. All tests revealed NPs with spherical and hexagonal disc shapes, as verified by XRD and TEM analyses. Alkane, amide, carbonate, alcohol, amine, and carboxylic acid were shown to be effective capping agents in these investigations. Furthermore, these synthesized NPs were also proved efficient in various biomedical applications like antimicrobial, anti-cancer, anti-diabetic, and antioxidants [20].

*Aquilegia pubiflora* is a medicinally valuable herb, which belongs to the *Ranunculaceae* family and is widespread in the Himalayas of India, northern Pakistan, and Afghanistan. This herbaceous plant is commonly called as hairy flowered columbine or Himalayan columbine but known locally as Thandi buti or Domba [22]. This species possesses many important pharmacological and medicinal properties including astringent, dyspepsia, cardiotonic, antiasthmatic, antipyretic, stimulant, and antijaundice. This plant's dried roots have been used to cure eye disorders, snakebites, homeopathy, inflammation, and toothaches and particularly for the nervous system [23]. A recent study found that the methanolic extract of *Aquilegia pubiflora* exhibits effective erythroid induction activity, indicating that this plant might be a source of fetal hemoglobin producing phytocompounds and could be utilized to treat *β*-thalassemia [24]. Moreover, *Aquilegia pubiflora* was mainly used for the treatment of influenza, skin burns, wound healing, jaundice, and gynecology, circulatory, and cardiovascular disease [25].

Here, we disclose the bio-assisted synthesis of ZnO-NPs through an ecofriendly approach using aqueous extracts of *Aquilegia pubiflora* as an efficient oxidizing/reducing and capping agent. The biosynthesis of ZnO-NPs has already been reported; however, their diverse biological properties including anti-Alzheimer's, antidiabetic, antiaging, and anticancer activities have been less exposed. The aim of the present study is therefore to investigate the biological effects of synthesized ZnO-NPs [7]. HPLC, FTIR, XRD, SEM, DLS, Raman, and PL were used to characterize the ZnO-NPs as we previously reported [26, 27]. The well-characterized ZnO-NPs were examined for their biological activities, including anti-inflammatory activity, anti-aging, anti-diabetic, and antioxidant activities. The anti-Alzheimer and anti-diabetic effects of ZnO-NPs were also screened, and the *in vitro* cytotoxic potential against cancer cell was examined for their possible application in the biomedical field.

## 2. Materials and Methods

### 2.1. Plant Identification and Extraction

The leaves of *Aquilegia pubiflora* were collected in the Swat area of Pakistan and determined to be disease-free and healthy. Plant identification and verification were carried out by Professor Mushtaq Ahmad of the Herbarium of the Department of Plant Sciences at Quaid-i-Azam University in Pakistan. The collected leaves were cut into tiny bits and properly washed with tap water to remove contaminants and dust spores. The cleaned leaves were stored in a closed room (at 25°C) for around 7 days to dry. In a sterile Willy mill, the dried leaves were crushed into a fine powder. To prepare the extract, 30 g of fine powder was mixed with 200 mL distilled water in a 500 mL flask, sonicated for 10 minutes, and maintained in a shaking incubator at 200 rpm and 37°C for two days. To get rid of any leftovers, the extract was filtered twice with nylon paper and then three times with Whatman No. 1 filter paper.

### 2.2. Biosynthesis of ZnO Nanoparticles

For the green synthesis of ZnO-NPs, the conventional process from Thema et al. was followed, with slight modifications to the extract and salt concentrations [28]. Before and after dissolving the aqueous extract with the precursor salt, UV and pH were monitored. 100 mL of extract was mixed with 6.0 g of zinc acetate dihydrate salt and swirled for 2 hours at 60°C on a magnetic stirrer. Only after reaction occurs, the temperature is lowered for 10 minutes at 25°C upon being centrifuged at 10,000 rpm (HERMLE Z326 K). The solution was separated, and the leftover pellet was rinsed multiple times with sterile water before being placed on a clean Petri plate and dried in the oven at 80°C. The dried materials were ground to a powdered form in a clean grinder and pulverized at 500°C for 2 hours to remove any impurities. For eventual physical characterization and biomedical uses, the annealed powder was put in a sealed sample vial, labeled, and stored.

### 2.3. Characterization of Biosynthesized ZnO-NPs

As previously reported, the morphological, structural, and vibrational physiognomies of obtained ZnO-NPs were investigated utilizing a variety of characterization methods such as HPLC, FTIR, XRD, SEM, DLS, Raman, and PL [27]. HPLC and Fourier transform infrared spectroscopy (FTIR) (400–4000 cm^−1^) were used to identify phytochemicals and associated functional groups on ZnO-NPs. XRD was used to verify the good crystallinity, phase recognition, and homogeneity of ZnO-NPs (Model-D8 Advance, Germany). To test the stability of ZnO-NPs after processing, the dispersal sustainability of the particles in purified water with various pH values was visually analyzed. SEM was used to investigate the morphological characteristics of particles. DLS was used to identify the optimum charge and stability of ZnO-NPs. Photoluminescence (PL) and Raman spectroscopy were used to identify oxygen vacancies and vibrational modes [27].

### 2.4. Antidiabetic Assays

To explore the anti-diabetic ability of biosynthesized ZnO-NPs, both *α*-glucosidase inhibition and *α*-amylase bioassays were performed.

#### 2.4.1. *α*-Amylase Inhibition Assay

The *α*-amylase inhibiting action of ZnO-NPs was evaluated using the methodology of Zohra et al. with minor variations in NP concentrations [29]. For this test, a 96-well microplate was utilized, and 10 L of ZnO-NPs was added to each well, followed by 15 L of sodium phosphate buffer (pH 6.9), 25 mL of alpha-amylase, and 40 mL of starch solution. After 30 minutes of incubation at 50°C, the reaction was halted by adding 20 mL of 1 M HCl and 90 L of iodine solution. Like a control treatment, acarbose was utilized, while DMSO is being used as a negative control. A microplate reader was used to detect absorbance at 540 nm. The % inhibition of alpha-amylase by samples was determined using the following formula. The experiment was carried out in triplicate and twice.

(1) $$\begin{array}{c} \%\text{Enzyme inhibition}=\left(\frac{\text{Abs Sample}-\text{Abs negative control}}{\text{Abs blank}-\text{Abs negative control}}\right)\times 100. \end{array}$$

#### 2.4.2. *α*-Glucosidase Inhibition Assay

The anti-diabetic potential of ZnO-NPs was additionally evaluated utilizing previously reported protocol by Saratale et al. with minor changes in sample concentration [30, 31]. At pH 6.8, 50 mL of phosphate buffer and *p*-nitrophenyl alpha-D-glucopyranoside substrate solution was produced, and 100 mg of BSA was added. 10 mL of ZnO-NPs was preincubated with 250 L *α*-glucosidase (0.15 units/mL) at 37°C for 5 minutes. The master mixture was kept at 37°C for 15 minutes. After simply adding 2 mL of 200 mM Na_2_CO_3_ solution, the process was stopped. The absorbance of *p*-nitrophenol generated was measured at 400 nm using a UV-Vis spectrophotometer. Using the formula below, the % inhibition of *α*-glucosidase by samples was calculated. In the experiment, acarbose was used as a positive control and carried out in triplicate and repeated twice.

(2) $$\begin{array}{c} \%\text{Enzyme inhibition}=\left(\frac{\text{Abs Sample}-\text{Abs negative control}}{\text{Abs blank}-\text{Abs negative control}}\right)\times 100. \end{array}$$

### 2.5. Antioxidant Assays

#### 2.5.1. Total Antioxidant Capacity (TAC) Determination

The TAC potential of ZnO-NPs was calculated by a previously described methodology by Shah et al., with slight modifications in applied concentration [31, 32]. Using a micropipette, 100 L of ZnO-NPs was added to the Eppendorf tubes. Thereafter, Eppendorf tubes comprising ZnO-NPs received 900 mL of TAC reagent (0.6 M sulfuric acid, 28 mM sodium phosphate, and 4 mM ammonium molybdate in 50 mL dH_2_0). The solution was allowed to cool to room temperature after 2.5 hours in a water bath at 90°C. A spectrophotometer reader was used to quantify the reagent absorbance at 630 nm. The amount of ascorbic acid equivalent (AAE) per milligram of analyte was used to calculate TAC. The test was repeated three times in total.

#### 2.5.2. Total Reducing Power (TRP) Determination

The total reduction power of ZnO-NPs was tested using the approach outlined by Nazir et al. [32, 33]. 100 mL of ZnO-NPs was combined with 400 mL of C_6_N_6_FeK_3_ and 0.2 molar phosphate buffer (pH 6.6) in Eppendorf tubes and maintained for 30 minutes at 55°C in a water bath. The solution was centrifuged for 8 minutes at 1200 rpm with 400 mL of C_2_HCl_3_O_2_ added to each Eppendorf tube. The supernatant (140 mL) from each combination was poured into the wells of a 96-well plate containing 60 mL ferric cyanide solution. A spectrophotometer reader was used to quantify the reagent absorbance at 630 nm. The amount of ascorbic acid equivalent (AAE) per milligram of analyte was used to calculate TRP. The test was repeated three times in total.

#### 2.5.3. Free Radical Scavenging Assay (FRSA)

The established protocol of Ahmed et al. evaluated the potential free radical scavenging capability of ZnO-NPs using a previously reported protocol [34, 35]. To assess the antioxidant capacity of test samples, DPPH reagents at concentrations ranging from 12.5 mL to 400 mL were employed. A 96-well plate was filled with 10 mL of ZnO-NPs, and 90 mL of DPPH reagent was applied to each well containing ZnO-NPs. The positive control was ascorbic acid, while the negative control was DMSO. A microplate detector was used to measure the absorbance of the reaction mixture at 515 nm.

#### 2.5.4. ABTS Assay

The ABTS test, commonly identified as the Trolox antioxidant assay, was performed using the procedure of Faisal et al. with minor changes in applied concentrations [33, 36]. An ABTS solution for the reaction was made by combining 2.45 mM potassium per sulphate with 7 mM of ABTS chemical, subsequently 16 hours in the dark incubation. Upon mixing with test sample, the resulting tubes were incubated in the darkness for 15 minutes at 25°C. The absorbance of the test sample was measured at 734 nm using the Microplate Reader (BioTek ELX800). Negative and positive controls were employed, respectively, with DMSO and Trolox. The antioxidant potential of the samples was measured in TEAC, and the experiment was repeated three times.

### 2.6. Anti-Alzheimer's Activity

Inhibiting the enzymes butyrylcholinesterase (BChE) and acetylcholinesterase (AChE) is a possible therapeutic target for Alzheimer's disease. The inhibitory ability of ZnO-NPs against AChE and BChE enzymes was evaluated using a slightly modified Elman's technique in terms of NP concentration and dosages, as previously published by Imran et al. [37, 38]. From the test sample, a concentration scale ranging from 12.5 *μ*g/mL to 200 *μ*g/mL was used. In brief, ZnO-NPs are distributed in a phosphate-buffered saline solution (PBS). The ultimate enzyme concentration for AChE was 0.03 U/mL, and for BChE, it was 0.01 U/mL. The reaction mixture was supplemented with DTNB (0.00022 M), acetylcholine iodide (ATchI; 0.0005 M), and butyrylcholine iodide (BTchI; 0.0005 M) produced in filtered water at 8°C. Galantamine hydrobromide (Sigma; GI660) produced in methanol was employed as a positive control in the experiment, and the reaction mixture stripped of the test sample was used as a negative control. The anticholinesterase activity is established on the splitting of ATchI into AChE and BTchI into BChE, which results in the production of the yellow-colored products. Using a spectrophotometer, the absorbance was eventually measured at 412 nm. Galantamine and ZnO-NPs have estimations for percent enzyme activity and percent enzyme inhibition with a temporal shift in the absorption rate. The following formulae were used to compute the percent enzyme inhibition.

(3) $$\begin{array}{c} V=\frac{\Delta Abs}{\Delta t},\\ \text{Inhibition }\left(\%\right)=100–\text{Enzyme activity }\left(\%\right),\\ \text{Enzyme activity }\left(\%\right)=\left(\frac{V}{V_{\max}}\right)\times 100. \end{array}$$

### 2.7. Anti-Inflammatory Activities

#### 2.7.1. Against COX-1 and COX-2

ZnO-NPs were evaluated for their ability to inhibit COX-1 (Ovine Kit 701050) and COX-2 (Human Kit 701050). As a positive control, ibuprofen 10 M was used, and arachidonic (1.1 mM) was used as a substrate. Both COX peroxidase components were measured in accordance with the kit's manufacturer's instructions. The test was carried out on a 96-well plate in triplicate and was repeated twice.

#### 2.7.2. Against 15-LOX

ZnO-NPs were tested for their ability to inhibit 15-LOX (760700 kit, Cayman France). 100 M NDGA was employed as a positive control, whereas 10 M C_20_H_32_O_2_ was used as a substrate. Hydroperoxides are formed as a result of lipooxygenation, and their concentration was measured using a 15-lipooxygenase standard in 10 mM Tris-HCl buffer at 7.4 pH filter supplied with the kit. In a 96-well plate, ZnO-NPs and enzyme are mixed together and incubated for 5 minutes. The 5-minute incubation was followed by 15-minute incubation after the addition of the substrate and a 5-minute incubation period after the addition of the chromogen. Using a Synergy II reader, the absorbance was measured at 590 nm (BioTek Instruments, Colmar, France).

#### 2.7.3. Against Secretory Phospholipase A2 (sPLA2)

An assay kit (10004883, Cayman Chem., France) was used to test the inhibitory ability of ZnO-NPs against sPLA2. 1.44 mM diheptanoyl thio-PC was used as a positive control, while 100 M thiotheramide-PC was used as a substrate. The cleavage of the diheptanoyl thio-PC ester produces free thiols, which were detected using DTNB at 420 nm in a 96-well microplate.

### 2.8. Antiaging Assay

#### 2.8.1. Anti-AGE Formation Activity

The previously reported protocol of Kaewseejan et al. was used to assess the inhibitory potential of vesperlysine AGEs and pentosidine AGE production [39]. 0.5 M glucose solution and 0.1 M PBS containing 0.02 percent (*w*/*v*) sodium azide were used to make BSA solution. ZnO-NPs were mixed with a 20 mg/mL BSA solution. The reaction mixture was kept at 37°C for five days in the dark. The fluorescence was estimated and quantified using a Versa Fluor fluorometer from Bio-Rad in France, with a 410 nm emission wavelength and a 330 nm excitation wavelength.

#### 2.8.2. Tyrosinase Assay

The tyrosinase test was performed using 5 mM L-DOPA, as previously described by Chai et al. [40]. L-DOPA diphenolase substrate was combined with 10 mL ZnO-NPs and sodium phosphate buffer (50 mM, pH 6.8). The final volume of the reaction mixture was raised to 200 mL by adding 0.2 mg/mL mushroom tyrosinase solution. As a control, extraction solvent in place of the tested ZnO-NPs was employed. At 475 nm, a microplate machine was used to track the reaction activities. The tyrosinase impact on ZnO-NPs was reported as % inhibition compared with matching control.

#### 2.8.3. Elastase Assay

Porcine pancreatic elastase was used in the elastase inhibition test (Sigma-Aldrich). The substrate used in the test was N(AAAVPN). The relative conversion of substrate into *p*-nitroaniline release at 410 nm was used to quantify the reaction traces using a microplate reader, following the approach of Wittenauer et al. [41].

#### 2.8.4. Hyaluronidase Assay

The potential of ZnO-NPs to inhibit hyaluronidase was tested using a technique established by Kolakul et al. [42]. A solution containing 0.03 percent (*w*/*v*) hyaluronic acid and 1.5 units of hyaluronidase was employed as a substrate. The undigested form of hyaluronic acid precipitated in an acid albumin solution (0.1 percent (*w*/*v*) BSA). The optical density (OD) at 600 nm was measured using a spectrophotometer. In contrast to the control, the antihyaluronidase potential was expressed as a percentage inhibition.

#### 2.8.5. Collagenase Assay

The procedure from Wittenauer et al. was used, with a small change in applied concentration [41]. FALGPA obtained from Sigma-Aldrich functioned as a substrate. The reduction in FALGPA absorbance was measured at 335 nm using a microplate reader over a period of 20 minutes. The trial was conducted in triplicates, and anticollagenase activity was expressed as a percentage inhibition compared to the control.

### 2.9. Cytotoxicity against the HepG2 Cell Line

HepG2 cells (ATCC HB-8065) were grown in DME medium with 100 g/mL streptomycin, 10% FCS, 100 U/mL penicillin, and 2 mM L-glutamine and incubated at 37°C in a humidified CO_2_ incubator with 5% humidity. 0.5 mM trypsin/EDTA was used to harvest the cells at 80-90 percent confluence. The cytotoxic potential of ZnO-NPs against HepG2 cells was determined using MTT tetrazolium dye. Preseeded HepG2 cells (>90% viability; 1 × 10^4^ cells/well) were treated for 24 hours in a 96-well plate with 200 *μ*g/mL test samples and incubated in a 5 percent humidified CO_2_ incubator. After a 24 h incubation period, 10 mL of MTT dye (5 mg/mL) was added to each well and incubated for 3 h. After that, the insoluble formazan was dissolved in a 10% acidified SDS solution. The cells were then incubated overnight. The plates were examined at 570 nm using a microplate reader (Platos R 496, AMP). As a control, we utilized untreated HepG2 cells (NTC) and doxorubicin.

(4) $$\begin{array}{c} \%\text{Viability}=\frac{\text{Absorbance of sample}-\text{Absorbance of sample control}}{\text{Absorbance of NTC}-\text{Absorbance of media }}\times 100. \end{array}$$

Optical density of treated samples and NTC was measured at 570 nm.

## 3. Results and Discussion

### 3.1. Synthesis and HPLC Analysis of Green ZnO-NPs

*Aquilegia pubiflora* leaf extract has been employed as a reducing and stabilizing agent in the production of multifunctional ZnO-NPs in recent study. The genus *Aquilegia* is a member of the *Ranunculaceae* family, which comprises around 60 plant species used for a variety of medicinal purposes across the world, mostly in South Asia. Among the medicinally important phytochemicals identified in these plants are ferulic acid, *β*-sitosterol, apigenin, aquilegiolide, magnoflorine, berberine, caffeic acid, *p*-coumaric acid, genkwanin, glochidionolactone A, and resorcylic acid [27, 43]. These phytochemicals, such as phenolics and flavonoids, may have played an important role in the formation of stable nanoparticles. HPLC was used to produce and quantify the fundamental phytochemicals responsible for the reduction and effective capping of synthesized NPs. Eight constituents were identified and measured, including four hydroxycinnamic acid derivatives (sinapic acid, ferulic acid, chlorogenic acid, and *p*-coumaric acid) and four flavonoids (orientin, vitexin, isoorientin, and isovitexin). Hydroxycinnamic acids and flavonoids are phenolics that are produced through the shikimic acid pathway and are involved in a range of biological processes in plants [27, 31]. Orientin and chlorogenic acid both protect plants against stresses and have a wide range of biological activities, including antifungal, anticancer, antidiabetic, antibacterial, antioxidant, anti-inflammatory, and hepatoprotective effects [44, 45]. According to the findings of earlier studies, flavonoids and hydroxycinnamic acid derivatives can be found on the surface of nanoparticles [27, 32, 46]. These plant active chemicals have a role in ZnO-NP capping. White ZnO-NP powder was produced after washing, drying, grinding, and calcination operations. The fine powder was collected and kept as ZnO-NPs in an airtight glass container before being employed for physicochemical and morphological evaluation, as well as biological applications [27].

### 3.2. Physicochemical and Morphological Characterization

**Figure 1:**
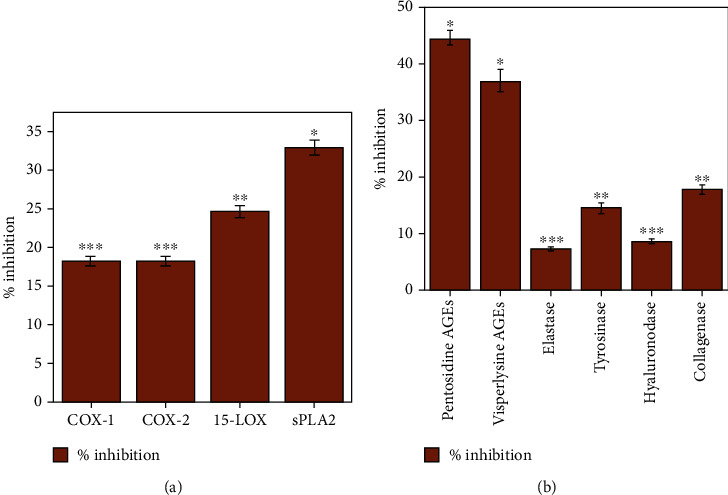
(a) Anti-inflammatory potential of synthesized ZnO-NPs with respect to their control value (68.39 ± 3.17). (b) Antiaging potential of ZnO-NPs with respect to their control value (74.83 ± 3.92). ^∗∗∗^Highly significant, ^∗∗^slightly significant, and ^∗^nonsignificant difference from control at *P* < 0.05 by one-way ANOVA in the column. Values are mean ± SD of triplicate.

**Figure 2:**
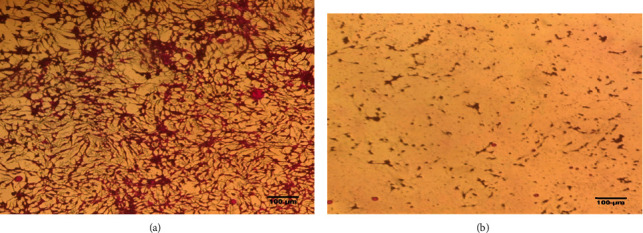
(a) Characteristic pictures of nontreated cells. (b) ZnO-NP-treated cells.

**Figure 3:**
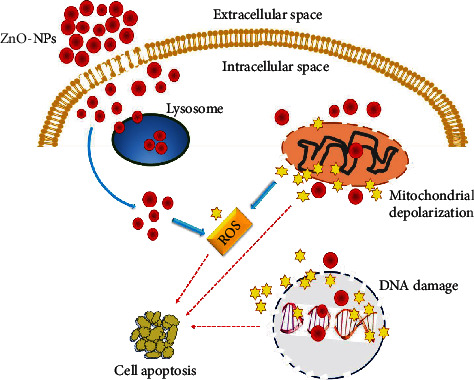
The proposed mechanism of ZnO-NP-mediated cytotoxicity in cancerous cell. NP accumulation and dissolution on the plasma membrane further lead toward lysosome and exert oxidative stress causing generation of ROS. Active ROS causes mitochondrial depolarization and DNA damages finally resulting in cell apoptosis.

The XRD pattern of synthesized ZnO-NPs showed the presence of pure and crystalline nanoparticles, with high diffraction peaks observed at various stages, i.e., 69.47°, 68.23°, 66.83°, 62.68°, 56.31°, 47.39°, 36.36°, 34.34°, and 36.36°, referring to different Miller indices, respectively (201), (212), (200), (103), (102), (101), (002), and (100) as shown in Figure 1S(A). The indexing of ZnO with a mean size of 19.58 nm supports the normal hexagonal wurtzite structure (JCPDF file no. 00-036-1451) [47, 48]. The FTIR spectra of the synthesized nanoparticles were estimated in the spectral range of 400–4000 cm^−1^ as shown in Figure 1S(B). The main absorption peaks were identified in the range of lower wavenumbers. The broad band observed at 3100 cm^−1^ corresponds to the O-H stretching mode of the hydroxyl group. The highest strength at 1459^−1^ in the protein amide connection indicated amine (-NH) vibration stretch. The prominent bands detected at 1028 cm^−1^ and 1384 cm^−1^ represented alcohols, phenolic compounds, and C-N stretching vibrations of aromatic amines in biomolecules. Zn-O refers to a distinct band that can be seen at 863 cm^−1^ [49–51]. The surface shape and particle size of green-synthesized ZnO-NPs are determined by scanning electron microscopy, as illustrated in Figures 2S(A) and 2S(B). The micrograph showed that, with some degree of aggregation, the particles exhibit a spherical structure. In previous research, such morphologies have also been identified. Furthermore, using the ImageJ program, the average particle size of the nanoparticles was 34.23 nm [52, 53]. The dispersion power of ZnO-NPs in deionized water at pH 2, pH 7, and pH 12 was investigated. It was noted that even after 24 h of sonication, ZnO-NPs demonstrated excellent dispersion at neutral pH. Moreover, at neutral pH, the dispersion effect was also enhanced by a highly negative zeta potential value [54]. The zeta potential and particle size distribution were investigated using the Malvern Zetasizer, as shown in Figures 3S(A) and 3S(B). The colloidal stability of particles is determined by the zeta potential ∣*ζ*∣, which is a common estimate of the surface load. Suspensions featuring ∣*ζ*∣ 15 mV are normally graded as stable colloids. The calculation also improves the stable dispersion potential of biogenic ZnO-NPs at pH 7 in distilled water. The negative surface charge on particles provides particle dispersion stability and prevents aggregation due to the high binding affinity of the extract compounds on metallic ions. In addition, calculations of the size distribution showed the average particle size to be 131 nm. The larger ZnO-NPs detected by DLS are due to the technique's bias against measuring larger particles (even aggregate) [54, 55]. Photoluminescence (PL) analysis of engineered nanomaterials is significant because it gives important information on the quality of the synthesized materials. Excitation wavelengths ranging from 500 nm to 900 nm were used to measure the PL. Characteristic peaks, referred to as trap state or deep state emission, were found at 510 nm, 552 nm, and 690 nm, as shown in Figure 4S(A). There are three possible charge states for oxygen vacancies in ZnO: neutral, single ionized, and double ionized. While the presence of oxygen is responsible for green oxygen emissions from ZnO-NPs, the rise in oxygen vacancies is linked to high PL emissions [56, 57]. Figure 4S(B) shows the Raman spectra of biosynthesized nanoparticles, which revealed typical peaks at 435 cm^−1^ and 572 cm^−1^ [27, 58].

### 3.3. Antidiabetic Activity

Diabetes mellitus (DM) is a metabolic condition characterized by hyperglycemia that persists. It is caused by a lack of insulin synthesis or a lack of insulin sensitivity in the body's cells [59]. The International Diabetes Federation (IDF) study (2017) estimated that 425 million adults have diabetes, with that number expected to rise to 629 million by 2045 [60]. Lowering postprandial hyperglycemia is one of the important clinical approaches to diabetes care. This can be accomplished by inhibiting two essential digestive tract enzymes that hydrolyze carbohydrates, i.e., alpha-amylase and alpha-glucosidase [59]. Various doses of ZnO-NPs ranging from 12.5 *μ*g/mL to 200 *μ*g/mL were tested in the assay for *α*-amylase and *α*-glucosidase inhibition. Our findings suggest that ZnO-NPs have a mild inhibitory effect on *α*-amylase and *α*-glucosidase activity, which is consistent with previous research (Table 1) [61]. At the highest concentration of 200 *μ*g/mL, the maximum inhibition of *α*-amylase was determined to be 310.24, whereas the maximum inhibition of *α*-glucosidase was calculated to be 22.691.23. Overall, both enzymes' % inhibition increased in a dose-dependent manner; however, *α*-amylase showed considerably greater inhibition as compared to *α*-glycosidase, while considering the comparable inhibitory effect of both enzymes. Hence, ZnO-NPs synthesized from *Aquilegia pubiflora* have moderate antidiabetic properties; this could be due to the presence of some active compounds that inhibit diabetes-related enzymes.

^∗∗∗^Highly significant, ^∗∗^slightly significant, and ^∗^nonsignificant difference from control at *P* < 0.05 by one-way ANOVA in the column. The values represent the mean ± SD of three replicates.

### 3.4. Antioxidant Activity

A variety of test methods have been devised and employed due to the relevance of antioxidants in protecting natural and man-made materials. Methods based on inhibited autoxidation, oxygen consumption kinetics, or the formation of hydroperoxides and primary oxidation products are among them. Secondary oxidation products (e.g., carbonyl compounds) were also determined analytically [62]. However, most test methods do not involve substrate autoxidation. For this purpose, we have used four different antioxidant assays to gain a better understanding of test samples' antioxidant potential in terms of secondary oxidation products, primary oxidation products, and oxygen consumption kinetics [63]. Because the radical is stable and does not need to be generated as in other scavenging assays, the DPPH method is the most valid, easy, accurate, sensitive, and cost-effective method for evaluating the scavenging activity of antioxidants in fruits, vegetables, juices, extracts, and extract-mediated nanomaterials [62]. The results are very repeatable and comparable to ABTS, TAC, ORAC, and FRAC, as well as other scavenging methods. Another strategy, which considers the antioxidant concentration and reaction time to reach the scavenging reaction plateau, has been found to be superior to other methods (considering only antioxidant concentration) [63].

For these assays, five separate concentrations, i.e., 200, 100, 50, 25, and 12.5 *μ*g/mL, were used and the results demonstrated that antioxidant potential of the ZnO-NPs was highly significantly upregulated shown in Table 2. Since aqueous extracts of *A. pubiflora* are available in this sample, it may be concluded that some of the phenolic compounds involved in capping of ZnO-NPs can quench the reactive oxygen species by acting as reducing/oxidizing agents. The test material's total antioxidant potential (TAC) is predicated on its conversion from Mo (VI) to Mo (V), and the greenish Mo (V)-phosphate complex had the fastest absorption at 695 nm. TAC assay reveals the quenching ability of the measured material compared to ROS species [64]. At a higher concentration of 200 *μ*g/mL ZnO-NP concentration, moderate (71.66 ± 1.14) TAC activity was observed while at lower concentration of NPs (12.5 *μ*g/mL) TAC activity was further decreased as 26.78 ± 1.22. The antioxidant potential of ZnO-NPs was further determined by engineered power reduction test (TRP). Redox-possessing agent can neutralize and absorb free radicals by transferring the ion from Fe^+^3 to Fe^+^2. ZnO-NPs with reducing strength are capable to reduce ferrous ions from ferric ions [65]. Reductants have the ability to reduce free radicals by splitting their chains and donating a hydrogen atom, and this reduction capacity is linked to antioxidant capacity [66].

TRP experiment indicated the maximum antioxidant potential (111.32 ± 1.24 *μ*g AAE/mg) for the 200 *μ*g/mL ZnO-NP tested concentration in terms of ascorbic acid equivalents in antioxidant activities. However, from overall antioxidant assays performed (TRP, TAC, DPPH, and ABTS) using different concentrations of ZnO-NPs, the highest antioxidant activity was noted for ABTS assay (178.45 ± 2.64) at 200 *μ*g/mL ZnO-NP tested concentration. The reducing strength of the ZnO-NPs in the performed assays (TRP, TAC, DPPH, and ABTS) was shown to be gradually decreased with the decrease of tested concentrations of NPs. The highest reduction capacity for TRP assay was revealed at 200 *μ*g/mL ZnO-NP concentration as 111.3 ± 2.44 *μ*g AAE/mg, and the least reduction potential was noted at 12.5 *μ*g/mL as 19.11 ± 2.6 *μ*g AAE/mg. Both spectrophotometric approaches, DPPH free radical scavenging and ABTS assays, are based on the quenching of stable-colored DPPH and ABTS radicals, indicating the ability to scavenge of antioxidants [67]. At a concentration of 200 *μ*g/mL ZnO-NPs, medium (23.5 ± 2.4) DPPH radical scavenging activity was observed while low radical scavenging potential (6.37 ± 1.82) was observed at 12.5 *μ*g/mL. Similarly, the highest ABTS activity (178.45 ± 2.64) was recorded at 200 *μ*g/mL ZnO-NP concentration and the lowest (45.73 ± 1.91) at 12.5 *μ*g/mL. Some of the antioxidant chemicals involved in the reduction and stabilization of ZnO-NPs throughout the manufacturing process could also be responsible for imparting antioxidant capabilities to ZnO-NPs, according to the findings.

^∗∗∗^Highly significant, ^∗∗^slightly significant, and ^∗^nonsignificant difference from control at *P* < 0.05 by one-way ANOVA in the column. Values are mean ± SD of triplicate.

### 3.5. *In Vitro* Anti-Alzheimer's Activity

Alzheimer's disease (AD) is a chronic neurological disease that accounts for 60% to 80% of dementia cases worldwide. The condition is characterized by a gradual decline in cognitive functions such as memory, executive and visual spatial functioning, personality, and vocabulary. In the United States alone, one person develops Alzheimer's disease every 65 seconds, which is alarming [68]. Cholinesterase inhibitors are new AD medicines accessible for people with any stage of the disease. For the successful inhibition of cholinesterase enzymes, a variety of synthetic and natural substances have been described. In tissue synapses or neuromuscular junctions, the enzymes catalyze the hydrolysis of acetyl choline (a neurotransmitter) into choline and acetic acid. Nanotechnology, a diverse field, is a potential hotspot for identifying various therapeutic strategies, including drug delivery across the blood-brain barrier in Alzheimer's disease. Efficient disintegration of mature fibrils and remarkable inhibition of the *β*-amyloid fibrillation mechanism have been suggested by *Terminalia arjuna*-dependent gold nanoparticles. In addition, gold nanoparticles have also been reported to be successful in inhibiting cholinesterase enzymes, suggesting that gold nanoparticles are neuroprotective [69]. Trehalose-functionalized gold nanoparticles disintegrate matured fibrils and inhibit protein aggregation [70]. *Bacopa monnieri*-based platinum nanoparticles commendably eradicate reactive oxygen species which tends to drop the ROS level in Parkinson disorder [71]. Selenium and other metal oxide nanoparticles are also used effectively for AD on *in vivo* and *in vitro* basis [72]. The decrease in acetyl choline levels leads to the development of Alzheimer's disease. The inhibitory reaction of two cholinesterase enzymes, butyrylcholinesterase (BChE) and acetylcholinesterase (AChE), was investigated using different biogenic ZnO-NP concentrations (AChE) [73]. The inhibitory response for both esterases was dose dependent, which was surprising. The most active concentration of ZnO-NPs was 200 *μ*g/mL, which inhibited AChE by 64.76 ± 1.69 and BChE by 67.49 ± 0.60. At 12.5 *μ*g/mL ZnO-NP concentration, the inhibitory response was 35.76 ± 1.01 for AChE and 27.51 ± 0.84 for BChE, respectively. Furthermore, no significant difference in percent inhibition was recorded against both enzymes at 25 *μ*g/mL and 50 *μ*g/mL concentrations of ZnO-NPs, with inhibition responses of 38.02 ± 1.03 at 25 *μ*g/mL and 40.02 ± 0.09 at 50 *μ*g/mL for AChE and 29.01 ± 0.94 at 25 *μ*g/mL and 30.01 ± 0.08 at 50 *μ*g/mL for BChE, respectively. Overall, ZnO-NPs were found to be highly active against both enzymes as indicated by their IC_50_ values of 102 *μ*g/mL and 125 *μ*g/mL for AChE and BChE. Overall results exhibited that biosynthesized ZnO-NPs have efficient anti-Alzheimer's activity and as shown by significant % inhibition at 200 *μ*g/mL concentration, against both enzymes, respective to control as shown in Table 3. The results of the present study exhibited that *A. pubiflora*-mediated ZnO-NPs have effective anti-Alzheimer's activity when applied in high concentrations, as shown by >65% inhibition against both enzymes. A previous study by Khalil et al. (2019) showed inhibition of several metallic NPs against AChE and BChE enzymes. Concentrations of biogenic metal oxide nanoparticles ranging from 1000 mg/mL to 62.5 mg/mL were examined. Surprisingly, both enzymes' responses to inhibition were found to be dose dependent. At 1000 mg/mL, biogenic lead oxide (PbO) nanoparticles were one of the most active samples, suppressing cholinesterases by 71% (AChE) and 67% (BChE). This was followed by cobalt oxide nanoparticles, which suppressed AChE and BChE by 70% and 68%, respectively. With IC_50_ values of 160.81 mg/mL and 261.67 mg/mL for AChE and BChE, respectively, biogenic iron oxide nanoparticles were the least effective [73].

^∗∗∗^Highly significant, ^∗∗^slightly significant, and ^∗^nonsignificant difference from control at *P* < 0.05 by one-way ANOVA in the column. Values are mean ± SD of triplicate.

### 3.6. *In Vitro* Anti-Inflammatory Activity

Inflammation is an automated response exhibited by the body's immune system, against various bacteria, irritants, damaged cells, and adverse stimuli. Anti-inflammatory activities have been documented both *in vivo* and *in vitro* for different metallic NPs and for many secondary metabolites. ZnO-NP HPLC analysis shows good capping of flavonoids such as orientin, isoorientin, vitexin, and isovitexin. These flavonoids can exert anti-inflammatory stress in numerous ways, such as cyclooxygenase inhibition with often selective activity on COX-1 vs. COX-2, phospholipase A2, and lipoxygenases (enzymes developing eicosanoids), thus reducing the concentration of leukotrienes and prostanoids that plays a main role in developing inflammation [74]. In certain types of inflammatory disorders, existing therapies are currently ineffective to treat and mitigate the progression of the condition or even to eradicate the signs and symptoms of inflammation [75]. For example, we know that NPs have an exceptional capacity to penetrate the microbial membrane, so this issue can be solved by increasing drug penetration into the active site of microbial infection. The aim of recent science for developing NPs is to avoid and manage inflammatory and contaminated sites for identification [76–78]. So, to check the anti-inflammatory potential of *A. pubiflora*-mediated ZnO-NPs, various *in vitro* pathways such as COX-1, COX-2, sPLA2, and 15-LOX inhibitions were tested. All the pathways produced the most productive outcomes for the inhibitory activity of all experiments conducted. sPLA2 was found to have the highest inhibitory activity (32.90 ± 0.99%), followed by 15-LOX (24.57 ± 0.79%), COX-1 (18.41 ± 0.54%), and COX-2 (18.23 ± 0.57%), respectively, as shown in Figure 1(a). Overall results described that *A. pubiflora*-mediated ZnO-NPs have showed efficient inhibition of two enzymes, sPLA2 and 15-LOX, of inflammatory processes.

### 3.7. *In Vitro* Antiaging Activity

This assay comprised a screening of *A. pubiflora*-mediated ZnO-NPs for anti-aging potential. A test sample (ZnO-NPs) at a fixed concentration of 200 *μ*g/mL was utilized to assess their in vitro potential to inhibit enzymes such as tyrosinase, elastase, collagenase, hyaluronidase, and AGEs. Collagenase, hyaluronidase, and elastase-like enzymes are responsible for the destruction of extracellular matrix components in enzymes. Deep wrinkles, skin tonus, and skin resilience losses are all caused by these enzymes [79–81]. Tyrosinase disorders are caused by the phenomenon of aging and are the main causative agents of malignant melanoma and freckles or melasma, such as pigment disorders [82]. Advanced glycation end products (AGEs) have been linked to aging and age-related disorders as a result of oxidative stress [83, 84]. Certain compounds that can deter these enzymatic processes or pathways are desirable and useful in cosmetic industries. According to several studies, SIRT-1 (a class III deacetylase) and radical aging theory have emerged as potent survival and oxidative stress management agents [85, 86]. In our recent study, ZnO-NPs produced with some phytochemicals have shown that these NPs are capable of being used as antiaging agents. Significant inhibitory activities of ZnO-NPs against pentosidine AGEs (up to 44.63 ± 1.26%) have been observed, followed by vesperlysine AGEs (up to 37.13 ± 1.99%). For collagenase (17.83 ± 0.81%) and tyrosinase (14.56 ± 0.89), ZnO-NPs had intermediate inhibitory effect. The lowest observed pronounced inhibitory effects observed for elastase and hyaluronidase were 7.51 ± 0.31 and 8.73 ± 0.37, respectively, shown in Figure 1(b). It has been explained from the above findings that ZnO-NPs have a good inhibitory capacity against two enzymes, AGEs of pentosidine and vesperlysine. Previous research has shown that ZnO may absorb UV radiation and protect the skin from additional damage, making it a potential antiaging agent. When coupled with TiO_2_, ZnO-NPs and green tea polyphenols show synergistic photoprotective effects on the skin, significantly reducing erythema [87]. ZnO is claimed to be an antiaging element in cosmetics and sunscreens since it is an active UVA/UVB-reflecting sunscreen ingredient that provides UVB protection of 75%. The anti-aging potential of ZnO-NPs and TiO_2_-NPs in sunscreens is owing to their capacity to form complexes with proteins and cause the creation of free radicals, hence triggering ROS and inhibiting many proteins involved in aging [88].

### 3.8. Anticancer Activity against the HepG2 Cell Line

Plant-derived compounds are a promising treatment option for hepatocellular carcinoma [89]. In our research, FTIR and HPLC analyses reported the presence of alcohols, phenolic groups, and C-N stretching vibrations of aromatic amines of biomolecules, on the surface of synthesized ZnO-NPs; these groups are considered physiologically significant towards treatment of various pathogenic diseases including cancer [90]. A scientific hotspot for cancer therapy is provided by the toxic nature of NPs, having effective anti-cancer potential [55, 90]. In this study, the cytotoxicity of ZnO-NPs against human hepatocytes (HepG2 cell line) has been examined. HepG2 cells showed reduced cell viability of 23.68 ± 2.1% after 24 h ZnO-NP treatment, as compared to respective controls as shown in Figure 2. Moreover, the phase-contrast microscopic images of HepG2 cells treated with ZnO-NPs showed prominent apoptosis of cells after for 24 h treatment. These results indicated the highly toxic effect of ZnO-NPs against human hepatocytes, as shown in Table 4. The three primary mechanisms responsible for the cytotoxic effect of ZnO-NPs include the breakdown of ZnO-NPs into Zn^+^2, reactive oxygen species (ROS) formation, and DNA damage [91–93]. Moreover, physical properties such as size, surface chemistry, and dose dictate the overall uptake, elimination, and antitumor properties of the ZnO-NPs [93]. Various studies have showed the anticancer and antiproliferative effect of ZnO-NPs, via upregulating the tumor suppressor genes and apoptotic genes, downregulating the antiapoptotic genes, inducing DNA fragmentation, ROS production, and caspase-3 enzyme in HepG2 cells [94–96]. Our results are also consistent with these previously published studies regarding anticancer potential of Au-NPs against HepG2 cells. Plant-derived Au-NPs typically cause cellular death by causing reactive oxygen species (ROS). ROS disrupts signal transduction pathways while also increasing cellular death [97, 98]. The CeO_2_-NP antiproliferative activities were also shown to successfully reduce the viability of the HepG2 cell line, with inhibition of 26.78%. The positive control doxorubicin and the negative control DMSO inhibited the HepG2 cells by 94.24% and 4.24%, respectively, as compared to the CeO_2_-NPs [46]. Hence, the marked anti-tumor activity against HepG2 cells showed an exciting potential of biosynthesized ZnO-NPs as promising anti-cancer agents.  Figure 3 shows the proposed mechanism of ZnO-NP-mediated cytotoxicity in cancerous cell; when ZnO-NPs get entry into the cancerous cell, they produced ROS species, disturbed mitochondrial membrane depolarization, and damaged DNA; all these events eventually leads to apoptosis or death of cancer cell.

Values are mean ± SD of triplicate.

## 4. Conclusion

This research work is the ongoing portion of the previously biosynthesized ZnO-NPs using aqueous extract of *Aquilegia pubiflora*, a well-known plant for its medicinal importance. The crystalline structure of the synthesized NPs has been confirmed by XRD analysis. The presence of phytochemicals in converting metallic ions to nanoparticles was investigated by FTIR and HPLC testing. SEM and Raman spectra determined morphology and vibrational modes, while apparent charge and steadiness were determined by DLS. The produced ZnO-NPs have shown good antioxidant and anti-Alzheimer capabilities. ZnO-NPs have also revealed a moderate inhibitory ability against alpha-amylase and alpha-glucosidase enzymes. Biosynthesized ZnO-NPs have shown an effective anti-Alzheimer's activity at a higher concentration and showed >65% inhibition against both AChE and BChE enzymes. It has been observed that biogenic ZnO-NPs are highly toxic to HepG2 cell lines as indicated by reduced cell viability of the HepG2 cell line after contact to ZnO-NPs. Moreover, ZnO-NPs have a good anti-aging property as demonstrated by their inhibitory capacity against two enzymes, AGEs of pentosidine and vesperlysine. Furthermore, *A. pubiflora*-mediated ZnO-NPs have showed efficient anti-inflammatory capacity as depicted by inhibition of sPLA2 and 15-LOX enzymes responsible for inducing inflammation. Our results concluded that the abovementioned ZnO-NPs could be considered for application in cosmetics, owing to their good anti-aging effect, and in treatment of various diseases including cancer, diabetes, Alzheimer's, and other inflammatory diseases, owing to their strong anti-cancer potential and efficient antioxidant properties. To explore its biomedical capabilities at both *in vitro* and *in vivo* levels, further research on ZnO-NPs is required.

## Figures and Tables

**Table 1:** *α*-Amylase and *α*-glycosidase potential at various concentrations.

Enzymes	Concentrations (*μ*g/mL)					
	+ Control	12.5	25	50	100	200
*α*-Amylase	88.63 ± 3.79	18.91 ± 0.73^∗∗∗^	22.49 ± 0.54^∗∗^	27.19 ± 0.63^∗∗^	28.36 ± 0.69^∗∗^	31 ± 0.24^∗^
*α*-Glucosidase	88.63 ± 3.79	16.31 ± 0.44^∗∗∗^	18.66 ± 0.59^∗∗^	21.43 ± 1.06^∗^	21.91 ± 1.19^∗^	22.69 ± 1.23^∗^

**Table 2:** Antioxidant potential of *A*. *pubiflora*-synthesized ZnO-NPs.

Conc. (*μ*g/mL)	TAC (*μ*g AAE/mg)	TRP (*μ*g AAE/mg)	ABTS (TEAC)	DPPH (% FRSA)	Ascorbic acid
200	71.66 ± 1.14^∗∗^	111.32 ± 1.24^∗∗^	178.45 ± 2.64^∗^	23.5 ± 1.38^∗∗∗^	210.29 ± 4.72
100	53.71 ± 1.32^∗∗^	86.71 ± 0.98^∗∗^	124.32 ± 1.99^∗^	18.11 ± 1.2^∗∗∗^	186.31 ± 4.19
50	42.43 ± 0.84^∗∗^	54.23 ± 0.92^∗∗^	92.63 ± 2.25^∗^	13.32 ± 1.74^∗∗∗^	132.20 ± 4.03
25	31.72 ± 0.71^∗∗^	32.87 ± 0.95^∗∗^	68.54 ± 2.21^∗^	7.0 ± 0.97^∗∗∗^	119.18 ± 2.83
12.5	26.78 ± 1.22^∗^	19.23 ± 1.13^∗∗^	45.73 ± 1.91^∗^	6.37 ± 1.82^∗∗∗^	84.71 ± 2.51

**Table 3:** Acetylcholinesterase (AChE) and butyrylcholinesterase (BChE) inhibition by ZnO-NPs at different concentrations.

Enzymes	Concentrations (*μ*g/mL)				
	12.5	25	50	100	200
AChE	35.76 ± 1.01^∗^	37.54 ± 0.54^∗∗^	40.89 ± 0.63^∗∗^	52.44 ± 0.69^∗∗^	64.76 ± 1.69^∗^
BChE	27.51 ± 0.84^∗∗^	27.69 ± 0.59^∗∗^	36.81 ± 1.06^∗^	49.73 ± 1.19^∗∗^	67.49 ± 0.60^∗^
+ Control	52.41 ± 2.19	58.72 ± 2.11	62.79 ± 2.44	81.83 ± 2.68	86.20 ± 2.91

**Table 4:** Anticancer potential of ZnO-NPs and plant extract toward the HepG2 cell line.

Test sample	% inhibition	% viability
ZnO-NPs	76.32 ± 1.69	23.68 ± 2.1
NTC	100 ± 2.27	0.00
Doxorubicin	97.35 ± 2.84	2.65
*Aquilegia pubiflora* extract	24.52 ± 0.49	75.48 ± 1.81

## Data Availability

The datasets used and analyzed during the current research work is available from the corresponding author on reasonable request.

## Supplementary Materials

Supplementary Materials We have provided supplementary material in a separate file. That file includes Figures 1S, 2S, 3S, and 4S as supplementary figures cited from our previous published article.
