# Supplementary material for: Plant-Based Synthesis of Zinc Oxide Nanoparticles (ZnO-NPs) Using Aqueous Leaf Extract of Aquilegia pubiflora: Their Antiproliferative Activity against HepG2 Cells Inducing Reactive Oxygen Species and Other In Vitro Properties
Source: Oxid Med Cell Longev. 2021 Aug 17;2021:4786227. doi: 10.1155/2021/4786227 (PMC8387193; doi:10.1155/2021/4786227)
Supplement: Supplementary Materials — We have provided supplementary material in a separate file. That file includes Figures 1S, 2S, 3S, and 4S as supplementary figures cited from our previous published article. [file 4786227.f1.docx]

**Plant-Based Synthesis of Zinc Oxide Nanoparticles (ZnO-NPs) Using Aqueous Leaf Extract of *Aquilegia pubiflora*: Their Anti-proliferative activity against HepG2 cells inducing Reactive Oxygen Species and other *In vitro* Properties**

Hasnain Jan^1*^, Muzamil Shah^1^, Anisa Andleeb^1^, Shah Faisal^2^, Aishma Khattak^3^, Muhammad Rizwan^4^, Samantha Drouet^5^, Christophe Hano^5^, Bilal Haider Abbasi^1*^,

^1^Department of Biotechnology, Quaid-i-Azam University, Islamabad 45320, Pakistan [hasnainjan@bs.qau.edu.pk](mailto:hasnainjan@bs.qau.edu.pk), [mshah@bs.qau.edu.pk](mailto:mshah@bs.qau.edu.pk) , [ansaandleeb097@gmail.com](mailto:ansaandleeb097@gmail.com), [bhabbasi@qau.edu.pk](mailto:bhabbasi@qau.edu.pk)

^2^Institute of Biotechnology and Microbiology, Bacha Khan University, KPK, Pakistan

[shahfaisal11495@gmail.com](mailto:shahfaisal11495@gmail.com)

^3^Department of Bioinformatics, Shaheed Benazir University Peshawar, KPK, Pakistan [aishama.khattak@yahoo.com](mailto:aishama.khattak@yahoo.com)

^4^Centre for Biotechnology and Microbiology, University of Swat, KPK, Pakistan [Muhammad.rizwan@uswat.edu.pk](mailto:Muhammad.rizwan@uswat.edu.pk)

^5^Laboratoire de Biologie des Ligneux et des Grandes Cultures (LBLGC), INRA USC1328 Université ď Orléans CEDEX2, France [samantha.drouet@univ-orleans.fr](mailto:samantha.drouet@univ-orleans.fr),[hano@univ-orleans.fr](mailto:hano@univ-orleans.fr)

^*^Correspondence

Hasnain Jan

Tel and Fax: +92-305-8464348

[hasnainjan@bs.qau.edu.pk](mailto:hasnainjan@bs.qau.edu.pk)


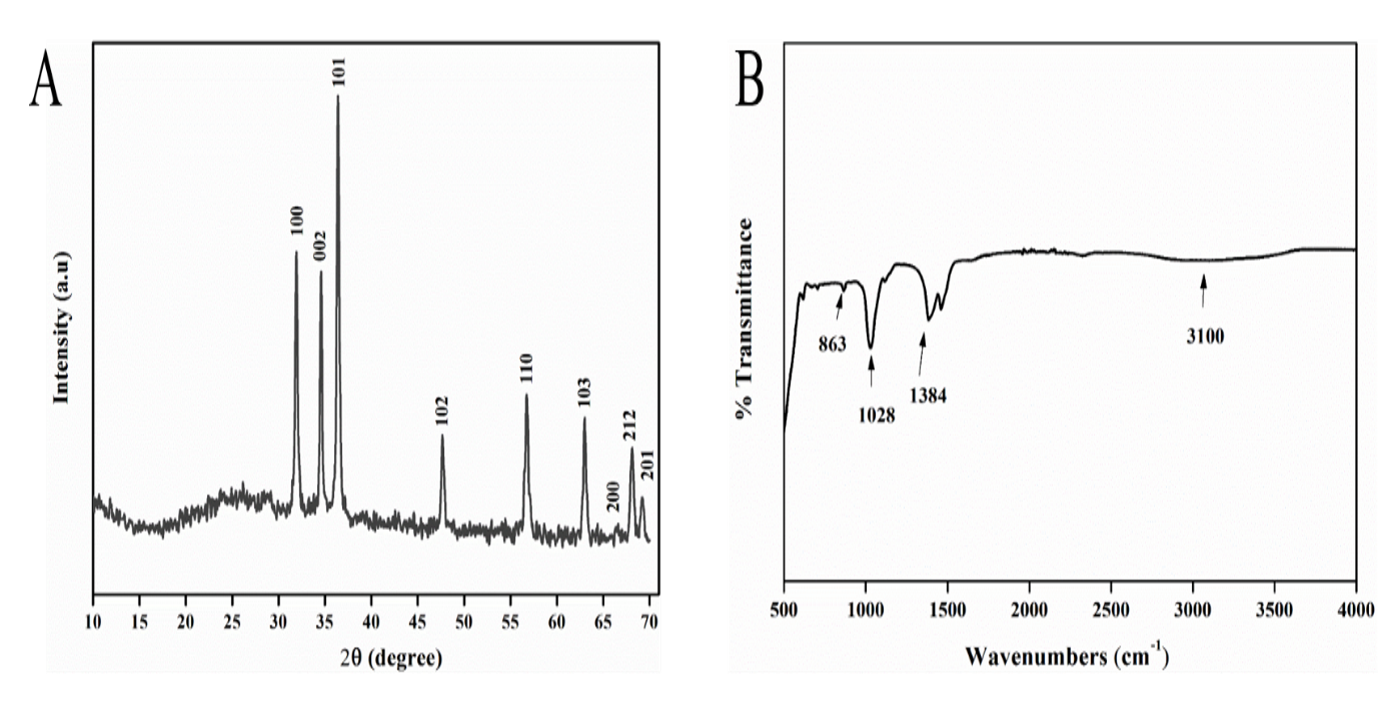


**Figure 1S:** A) X-ray diffraction (XRD) pattern of green-synthesized ZnO-NPs showing Bragg reflection at angle 2 theta. B) Typical FTIR spectra of ZnO-NPs. Published source [10.3389/fmats.2020.00249](https://ui.adsabs.harvard.edu/link_gateway/2020FrMat...7..249J/doi:10.3389/fmats.2020.00249). This figure is cited from our previous research article as it is the continuation section of our published work.

**
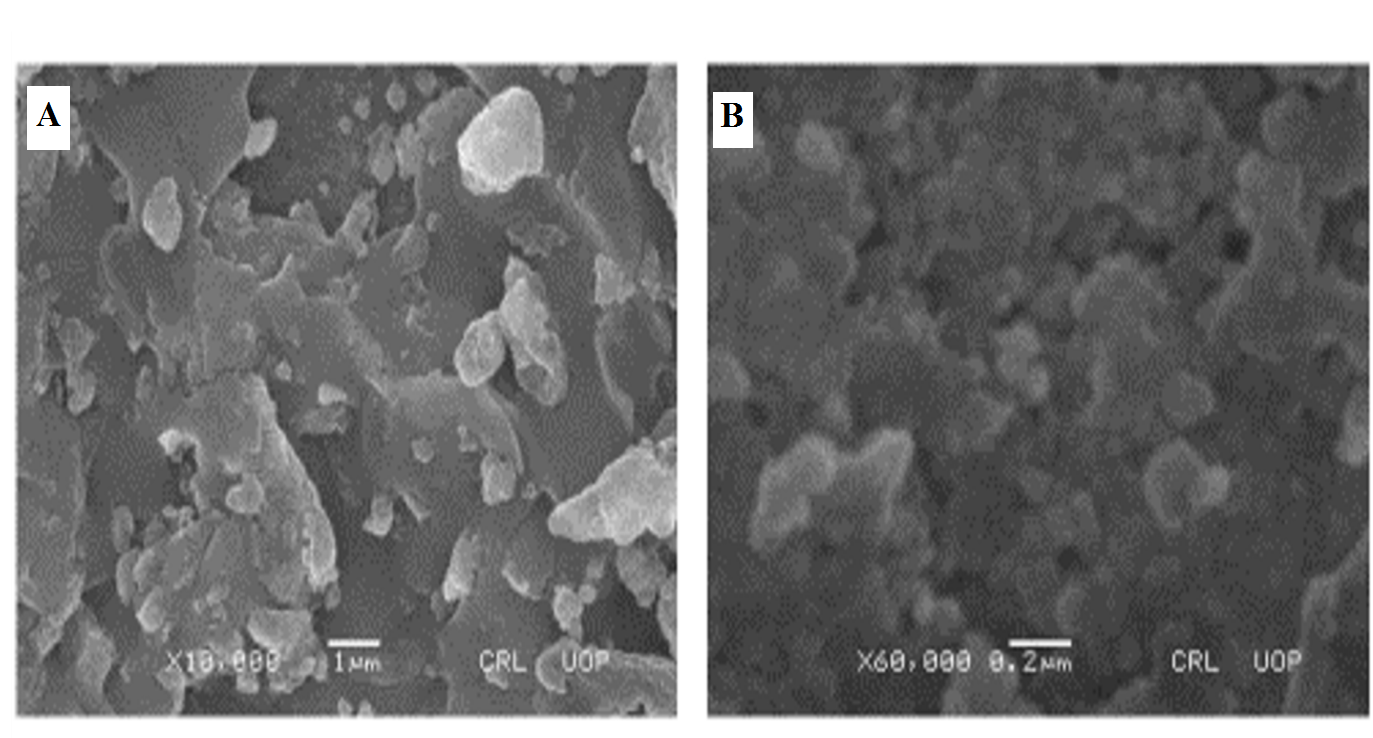
**

**Figure 2S:** A) Typical SEM micrograph of ZnO-NPs at 1 µm and B) at 200 nm showing elliptical morphology. Published source [10.3389/fmats.2020.00249](https://ui.adsabs.harvard.edu/link_gateway/2020FrMat...7..249J/doi:10.3389/fmats.2020.00249). This figure is cited from our previous research article as it is the continuation section of our published work.

**
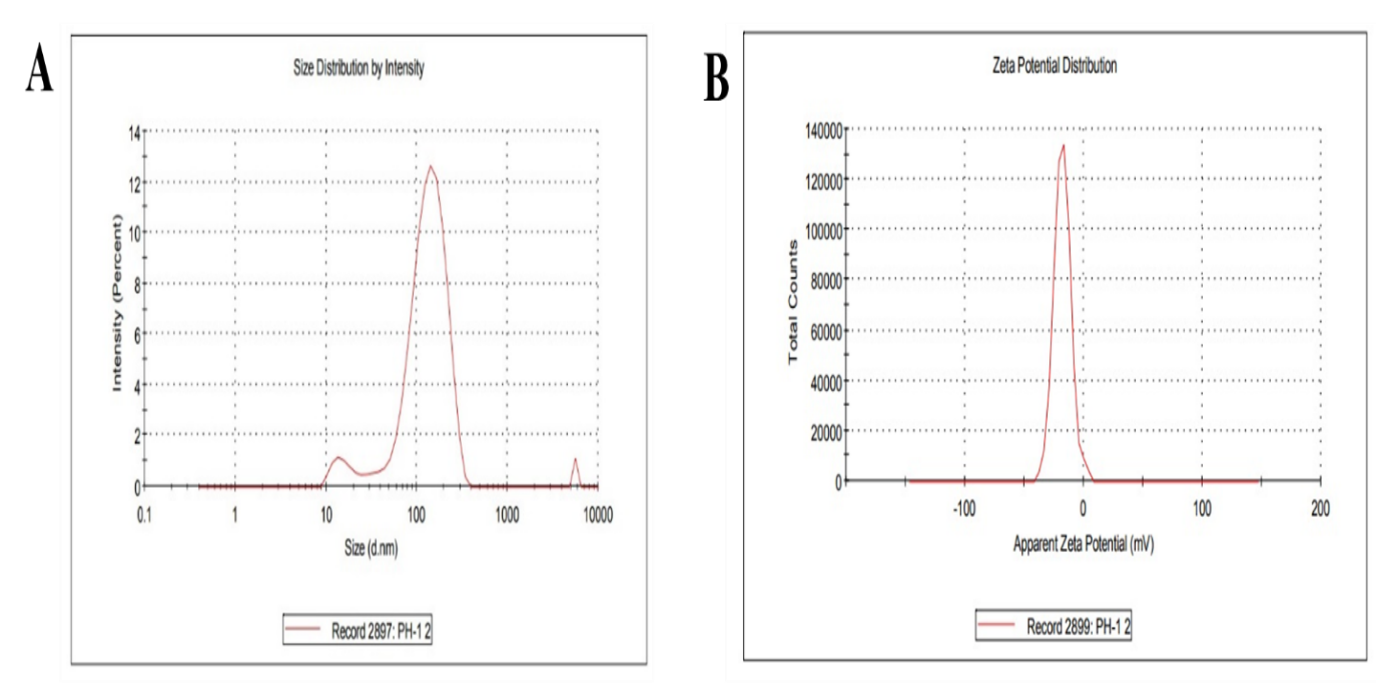
**

**Figure 3S:** A) DLS Size distribution, B) DLS Zeta Potential of ZnO-NPs.Published source [10.3389/fmats.2020.00249](https://ui.adsabs.harvard.edu/link_gateway/2020FrMat...7..249J/doi:10.3389/fmats.2020.00249). This figure is cited from our previous research article as it is the continuation section of our published work.


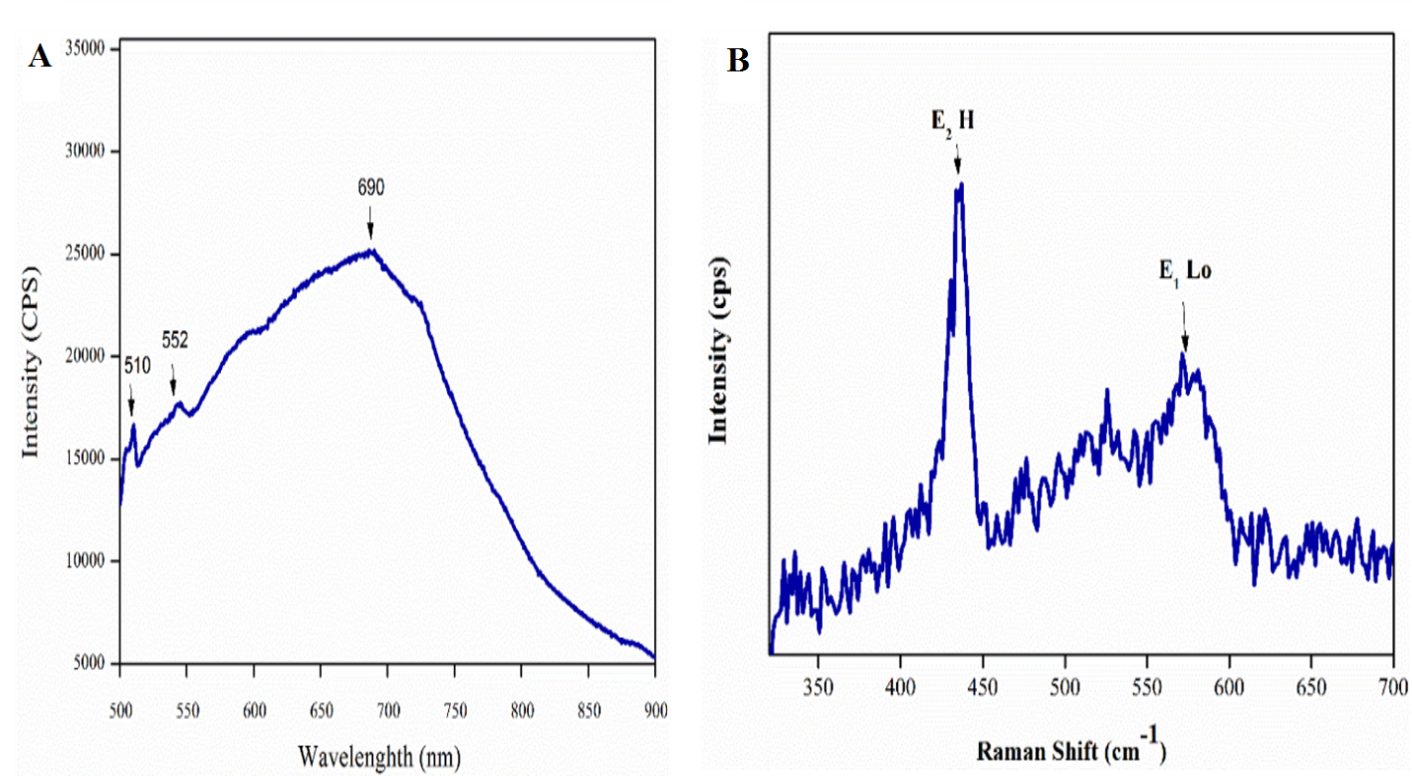


**Figure 4S:** A) Room temperature Photoluminescence analysis and B) Typical RAMAN spectra of ZnO-NPs. Published source [10.3389/fmats.2020.00249](https://ui.adsabs.harvard.edu/link_gateway/2020FrMat...7..249J/doi:10.3389/fmats.2020.00249). This figure is cited from our previous research article as it is the continuation section of our published work.
